# Pathophysiology, assessment, and treatment of pain in patients with pancreatic ductal adenocarcinoma

**DOI:** 10.1007/s00535-026-02423-3

**Published:** 2026-05-02

**Authors:** Mahya Faghih, Marko Damm, Charlotte Gutzler, Michael Hirth, Marie-Theres Kassik, Louise Kuhlmann, Patrick Michl, Søren S. Olesen, Anna E. Phillips, Vikesh K. Singh, Asbjørn M. Drewes

**Affiliations:** 1 Division of Gastroenterology, Department of Medicine, Johns Hopkins Medical Institutions, Baltimore, MD USA; 2 Department of Internal Medicine I, University Hospital Halle, Martin-Luther-University Halle-Wittenberg, Halle (Saale), Germany; 3 Department of Gastroenterology, Hepatology & Infectious Diseases, Heidelberg University Hospital, Heidelberg, Germany; 4 Department of Medicine II, Medical Faculty Mannheim, University of Heidelberg, Heidelberg, Germany; 5 Centre for Pancreatic Diseases & Mech-Sense, Department of Gastroenterology and Hepatology Clinical Institute, Aalborg University Hospital, Mølleparkvej, 9000 Aalborg, Denmark; 6 Division of Gastroenterology, Hepatology, and Nutrition, Department of Medicine, University of Pittsburgh School of Medicine, Pittsburgh, PA USA

**Keywords:** Abdominal pain, Pancreatic ductal adenocarcinoma, Pathophysiology, Neuroimmunology

## Abstract

Pain is one of the most frequent and debilitating symptoms associated with pancreatic ductal adenocarcinoma (PDAC). More than 60% of patients suffer from significant pain at diagnosis. The prevalence increases during the progression of the disease and is associated with anorexia, weight loss, and impaired social interactions. The pathophysiology of pain includes the combined effects of tumor growth and spread, perineural invasion, neuroimmune interactions, peripheral nerve remodeling, and central nervous system sensitization. Pain is additionally modulated by comorbid conditions, such as anxiety or depression, as well as treatment-related toxicity. Previous reports have used simple unidimensional scales to assess pain intensity, but as pain in PDAC is multidimensional, there is a need to develop new and robust instruments to assess pain. The treatment follows the World Health Organization three-step analgesic ladder. Non-opioid analgesics can be used to improve pain, but strong opioids are often used to relieve pain and suffering. When opioids are used, there should be a focus on the management of the side effects. Patients with anxiety and depression may benefit from treatment with selective serotonin reuptake inhibitors, serotonin/norepinephrine reuptake inhibitors, and tricyclic antidepressants that also have effects on pain. In some cases, more experimental drugs such as ketamine may be used. Celiac plexus neurolysis and local irradiation therapies are supplementary methods used to treat PDAC pain, but the response is unpredictable, and the durability is relatively short. The reduced survival associated with celiac ganglia injection, especially in patients with advanced disease, has also tempered enthusiasm.

## Introduction

Pancreatic ductal adenocarcinoma (PDAC) remains one of the most lethal malignancies worldwide, characterized by late diagnosis, limited therapeutic options, and poor overall survival [1, 2]. Despite advances in systemic treatment and supportive care, pain continues to be one of the most frequent and debilitating symptoms associated with the disease [1, 3]. More than 60% of patients suffer from significant abdominal or back pain at diagnosis, and the prevalence increases as the tumor progresses [3, 4]. The consequences extend well beyond physical discomfort: persistent pain is strongly associated with anorexia, weight loss, decreased mobility, sleep disruption, impaired social interactions, and diminished emotional resilience. Many patients describe pain as the most distressing aspect of their illness, significantly affecting autonomy and dignity [5]. Pain also has measurable prognostic implications, correlating with accelerated functional decline and reduced overall survival [6–8]. Despite this profound burden, pain remains under-recognized and undertreated in a substantial proportion of patients, highlighting the need for more precise assessment tools and mechanism-based therapeutic strategies [5].

The mechanisms contributing to pain in pancreatic cancer are complex, involving both nociceptive and neuropathic pathways [9]. Tumor invasion of peripheral nerves, the celiac plexus, splanchnic nerves, and surrounding retroperitoneal tissues is a hallmark finding and leads to persistent stimulation of visceral afferent fibers [10, 11]. At a molecular level, pancreatic tumors are known to produce high levels of neurotrophic factors, cytokines, and inflammatory mediators that induce neural remodeling, peripheral sensitization, and finally central sensitization within the spinal cord [12–16]. These biological processes underscore that PDAC-induced pain is not merely a consequence of mechanical tissue damage but results from a highly dynamic tumor–neural interaction.

Given this complexity, a thorough and multidimensional assessment of pain is essential. Beyond measuring intensity (e.g., via numerical or visual-analog scales), evaluation must consider pain quality (visceral vs. neuropathic), temporal patterns, functional impairment (nutrition, sleep, daily activities), psychological distress, and response to treatment. Psychological evaluation is also critical, as depression and anxiety can exacerbate pain and complicate analgesic management. Early identification and tailored intervention can prevent the transition from acute to chronic pain and reduce the need for escalating opioid therapy [17].

Pharmacological treatment remains the basis of pain management in PDAC and typically follows the World Health Organization cancer pain ladder [9, 18]. However, systemic therapy alone often provides incomplete relief, and adverse effects may limit tolerability, particularly in patients receiving intensive multi-agent chemotherapy. As a result, interventional strategies including celiac plexus neurolysis (CPN) have become an important component of multidisciplinary care [19–21]. Despite available therapeutic strategies, pain management in PDAC remains challenging. Many patients continue to experience significant and persistent symptoms, and treatment selection often relies on clinical experience rather than mechanistic understanding. Emerging research into neuroimmune signaling, tumor–nerve interactions, and pain-specific biomarkers has highlighted new potential targets for intervention. Precision-based approaches, integrating molecular insights with clinical assessment, may enable more individualized and effective pain management in the future.

This review summarizes current knowledge on pathophysiology, assessment, and treatment of pain in PDAC, highlighting the role of pharmacological and interventional strategies, and discusses future perspectives for improving PDAC-induced pain.

## Pathophysiology of pain in pancreatic ductal adenocarcinoma

The pathophysiology of pain in PDAC is multifactorial and potentially exacerbated by clinical factors including comorbid exocrine pancreatic insufficiency or psychological distress. Tumor growth, ductal obstruction, capsular stretch, and celiac plexus involvement generate visceral nociceptive input, and perineural invasion (PNI), neural remodeling, and neuroinflammation add a neuropathic component.

### Mechanical factors and local extension

**Fig. 1:**
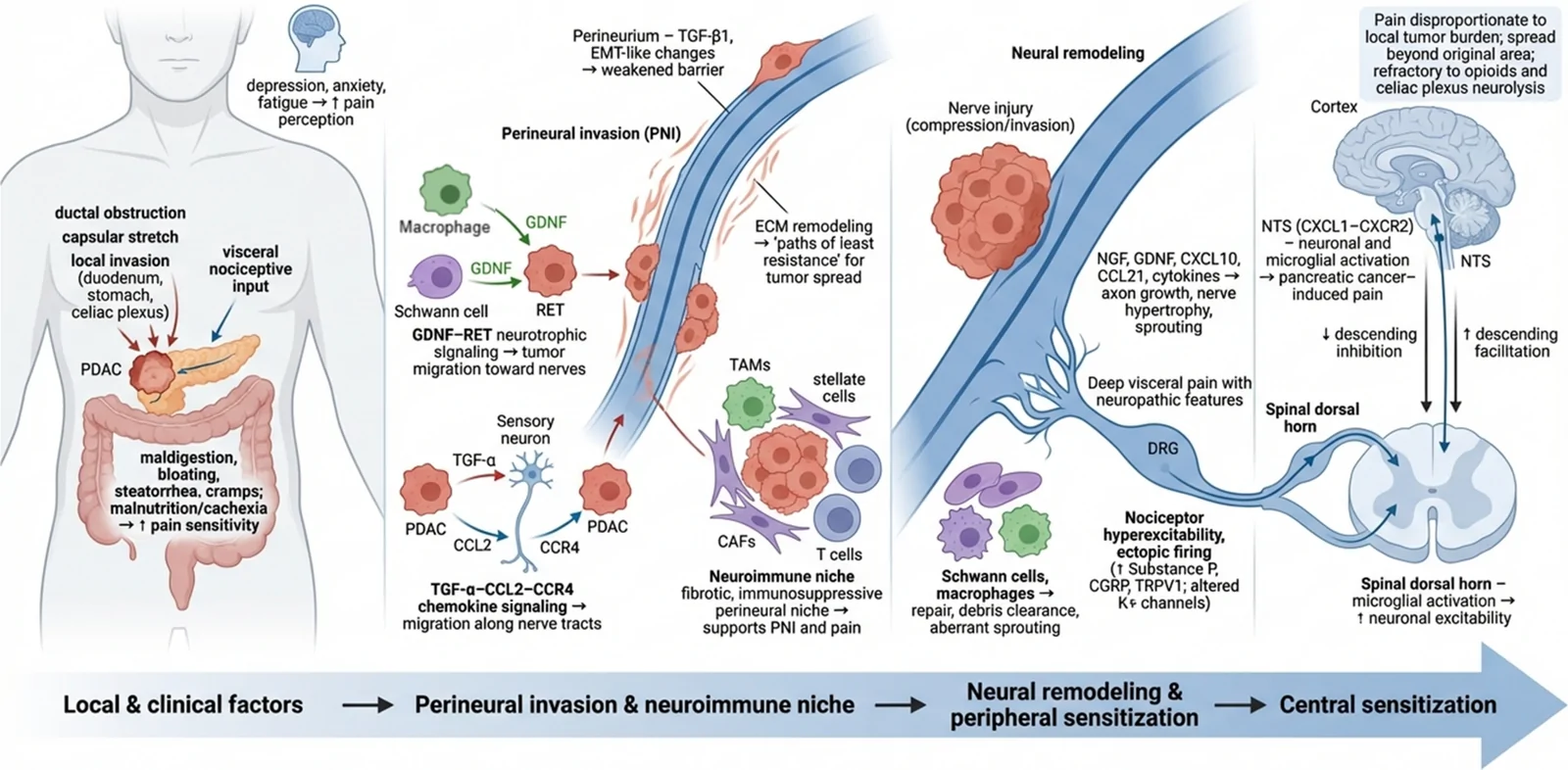
Integrated mechanisms of pain in pancreatic ductal adenocarcinoma. Pain in PDAC results from the interaction of local tumor-related factors, perineural invasion, and neuroimmune signaling, neural remodeling with peripheral sensitization, and central sensitization involving spinal and brainstem pathways, leading to persistent visceral and neuropathic pain. Blue structures depict larger nerve trunks or pathways, whereas red elements highlight individual tumor cells or representative neuronal/cellular components; differences in size are schematic and not drawn to anatomical scale. *PDAC* pancreatic ductal adenocarcinoma, *PNI* perineural invasion, *EPI* exocrine pancreatic insufficiency, *GDNF* glial cell-derived neurotrophic factor, *RET* rearranged during transfection receptor tyrosine kinase, *TGF-α* transforming growth factor-α, *TGF-β1* transforming growth factor-β1, *CCL2* C–C motif chemokine ligand 2, *CCR4* C–C motif chemokine receptor 4, *NGF* nerve-growth factor, *CXCL10* C–X–C motif chemokine ligand 10, *CCL21* C–C motif chemokine ligand 21, *TAMs* tumor-associated macrophages, *CAFs* cancer-associated fibroblasts, ECM extracellular matrix, *MMPs* matrix metalloproteinases, *DRG* dorsal root ganglion, *CGRP* calcitonin gene-related peptide, *TRPV1* transient receptor potential vanilloid 1, *CNS* central nervous system, *NTS* nucleus tractus solitarius

Tumor size, location (particularly head of pancreas lesions), and local extension can cause pancreatic and biliary duct obstruction and increased intraductal and capsular pressure [22]. Direct infiltration into adjacent organs and structures (such as the duodenum, stomach, mesentery, and celiac plexus) and peritoneal or retroperitoneal metastases can result in local inflammation, tissue stretching, and direct nerve involvement that can lead to pain (Fig. 1) [1].

### Perineural invasion and tumor–nerve crosstalk

PNI is one of the most characteristic histologic features of PDAC and is strongly associated with pain severity, opioid requirements, and worse prognosis in the clinical setting. In a case series of patients who underwent pancreatic head resection, the extent of PNI and intrapancreatic nerve density were seen to correlate with preoperative pain [6] and early recurrence [23, 24].

A key pathway is neurotrophic (nerve-growth factor) signaling involving glial cell-derived neurotrophic factor (GDNF) and its receptor, REarranged during Transfection (RET). Murine models have shown that cells surrounding the nerve (such as perineurial macrophages and Schwann cells) release GDNF, while PDAC cells increase their expression of the RET receptor [25–29] (Fig. 1). This combination acts like a “nerve attractant” where GDNF is the signal, and RET is the antenna. Together, they pull cancer cells toward nerves and help them grow into and along the nerve sheath. In murine PDAC models, blocking GDNF, RET, or the recruitment of GDNF-producing macrophages reduces PNI and reduces pain-like behaviors. Translational studies in humans have shown higher GDNF and RET expression in tumors with extensive PNI and more severe pain.

Chemokine and cytokine signaling further shapes this tumor–nerve interaction. Preclinical work shows that tumor-derived Transforming Growth Factor (TGF-α) can stimulate sensory neurons to release CCL2 (C–C motif chemokine ligand 2; a small cytokine that acts as a powerful chemoattractant for immune cells like monocytes), which then activates the receptor CCR4 (C–C motif chemokine receptor 4, a protein that acts as a cell surface receptor) on PDAC cells and enhances directed migration along nerve tracts [30, 31]. Interrupting this TGF-α–CCL2–CCR4 axis in mouse models reduces neural invasion and mechanical hypersensitivity. Increased expression of these chemokines and their receptors have also been reported in human PDAC, particularly in tumors with marked PNI [30, 31].

Schwann cells are also active players in this process. Tumor-derived factors including TIMP1 (Tissue Inhibitor of Metalloproteinase 1), vitamin D-binding protein (GC), and L1CAM (L1 tumor-associated molecule) drive Schwann cell dedifferentiation, migration, and secretion of additional neurotrophic mediators in vitro, and Schwann cell activation is consistently seen in nerves with PNI in vivo [30, 32–34]. Preclinical work suggests a metabolic partnership between Schwann cells and PDAC cells that supports tumor growth and perineural spread [30, 32–34]. Neuropathologic studies of human PDAC confirm Schwann cell proliferation and disorganization in areas of PNI and link these changes to pain [8, 35, 36].

At the structural level, factors released by PDAC such as TGF-β1 can change the cells that normally form the protective sheath around nerves (the perineurium). These cells adopt a more mobile, mesenchymal-like state (epithelial–mesenchymal transition), which weakens this barrier and makes it easier for cancer cells to enter and track along nerves [33, 34, 37]. In parallel, enzymes that break down the surrounding connective tissue and changes in cell-adhesion signaling promote degradation of the extracellular matrix, facilitating easier migration for cancer cells along neural planes [33, 34, 37]. These changes have been demonstrated in both in vivo PDAC models and human specimens with a high burden of PNI. Pancreatic stellate cells that express periostin and tumor-associated macrophages, which are enriched around nerves in PDAC, further remodel this area into a stiff, scar-like and immunosuppressed environment that promotes PNI and is associated with more severe pain in translational models [35, 36, 38].

### Neural remodeling and the tumor microenvironment

Quantitative studies of human PDAC specimens show increased nerve density, hypertrophy, and altered fiber composition compared to normal pancreatic tissue; these features correlate with higher pain scores, neuropathic pain descriptors, and poorer survival [8, 39]. Similar changes (nerve sprouting, hypertrophy, and disorganized innervation) have been reproduced in orthotopic PDAC models and are associated with symptoms of hyperalgesia and allodynia, suggesting the development of neural hypersensitivity occurring in conjunction with malignancy [8, 12, 39].

Tumor and stromal cells secrete neurotrophins (nerve-growth factor (NGF), GDNF), chemokines (CXCL10, CCL21), and inflammatory cytokines that drive axon growth and nerve hypertrophy [8, 12, 39, 40]. In murine PDAC, chemokines released from neurons and stromal cells attract tumor cells and induce nerve fiber hypertrophy; neutralizing these chemokines reduces nerve remodeling and pain behaviors [12]. Upregulation of neurotrophins’ signaling has also been reported in both experimental models and human PDAC tissue, particularly in painful tumors [40, 41].

Invasion and compression of nerves cause direct nerve injury, triggering Schwann cell proliferation, apoptosis, and macrophage-mediated clearance of debris [36]. This disordered repair process, seen in both animal models and human perineural lesions, leads to aberrant sprouting (misdirected growth of regenerating axons following nerve injury) and ectopic impulse generation. At the same time, changes in neurotransmitter and ion-channel expression [including increased substance P and CGRP and altered transient receptor potential vanilloid 1 (TRPV1) and potassium channel activity] have been observed in dorsal root ganglia and intrapancreatic nerves in PDAC models and are associated with heightened neuronal excitability and enhanced nociceptive transmission [42]. Clinically, these processes are thought to contribute to the combination of deep visceral pain and superimposed burning, shooting, or stimulus-evoked pain that many PDAC patients describe.

### Central nervous system sensitization and plasticity

Central nervous system sensitization represents an additional contributor to the pain pathophysiology in PDAC. Persistent nociceptive input from PNI and neural remodeling, together with systemic inflammation and stress, drives central sensitization, in which pain processing in the central nervous system becomes amplified, leading to heightened sensitivity to nociceptive input and more persistent pain [43].

In preclinical pancreatic cancer models, microglial activation has been demonstrated in brainstem and spinal regions involved in visceral pain processing, including the nucleus tractus solitarius (NTS) [43]. Chemokine signaling contributes to this activation; pharmacologic inhibition of this axis reduces microglial activation and alleviates PDAC-related pain behaviors [43]. These results align with broader cancer pain literature and are supported indirectly in PDAC by reports of elevated central inflammatory markers in patients with severe, refractory pain [44].

Central sensitization also involves disruption of descending modulatory pathways. Animal studies suggest that tumor-induced pain involves central neuroinflammatory changes, particularly activation of microglia in brainstem pain-processing centers through CXCL1–CXCR2 signaling, which amplifies nociceptive transmission and contributes to central sensitization [43]. Clinically, this is reflected in patients whose pain is out of proportion to local tumor burden, spreads beyond the original area, or remains refractory to opioids and regional procedures such as celiac plexus neurolysis.

Over time, ongoing pain leads to structural and functional changes in central networks. Experimental cancer pain models show cortical reorganization and altered connectivity within sensory and affective-cognitive pain circuits [45]. Although direct neurophysiological and imaging data in PDAC are limited, similar patterns in other chronic visceral pain conditions, together with clinical features like allodynia, sleep disturbance, fatigue, and mood changes, suggest that comparable central changes occur in PDAC [43, 45].

Taken together, clinical and preclinical data support a model in which perineural invasion, neuroimmune crosstalk, neural remodeling, and central sensitization form a connected pathway underlying PDAC‑related pain. Additional comorbid clinical conditions, such as exocrine pancreatic insufficiency and/or psychosocial distress related to underlying anxiety or depression, may contribute to the complexity of the pain experience. In addition, treatment‑related toxicity has the potential to further complicate pain in patients who experience this complication.

## Pain assessment

### Current pain assessment

Pain in PDAC has primarily been assessed using unidimensional intensity scales, such as the visual analog scale or the numeric rating scale. These tools capture the intensity, but not disease-specific features or temporal variation. Some studies include multidimensional scales such as the Brief Pain Inventory, which assesses not only pain severity, but also its interference with daily activities. However, it still lacks PDAC-specific features, such as postprandial exacerbation or neuropathic descriptors.

Despite guidelines recommending multidimensional evaluation as the foundation for sufficient pain management, most studies do not comply with these recommendations. In a multicenter cohort of 139 patients with advanced PDAC, nearly 90% experienced pain-related interference with daily function and mood [5]. Pain distress has also been shown to correlate strongly with psychological distress and with concurrent symptoms, such as appetite loss, fatigue, and sleep disturbance [46]. This emphasizes that PDAC pain is rarely an isolated symptom, but part of a broader biopsychosocial distress profile, where assessment should include aspects beyond pain intensity**. **Accordingly, routine assessments should document pain location and radiation, pain quality, temporal pattern including breakthrough pain, and aggravating and alleviating factors, as recommended in the current cancer pain guidelines [22, 47]**. **It should also capture functional interference with sleep, appetite, mobility, work, and daily activities, together with psychological distress such as depression, anxiety, catastrophizing, and sleep disturbance, all of which are associated with greater pain severity and interference in cancer populations [22, 47]. In PDAC, etiologic context is particularly important, because pain may reflect ductal obstruction, local invasion, or perineural involvement, and this distinction may influence whether pharmacological therapy alone is appropriate or whether earlier interventional approaches should be considered [22, 47, 48].

This is particularly relevant in PDAC, where the pain phenotype typically combines visceral, somatic, and neuropathic mechanisms, and assessment should be able to encompass characteristics specific to all of these different pain types. Patients often describe a deep, dull, or burning epigastric pain radiating to the back in a “belt-like” pattern [49, 50]. It thereby has several features similar to pain in other pancreatic diseases, such as chronic pancreatitis where multidimensional instruments to assess pain have been developed [51]. Generic cancer pain questionnaires often do not capture these characteristics. They do not differentiate between visceral and neuropathic qualities, do not record circadian fluctuation, and exclude neuropathic descriptors, such as burning or electric-shock sensation [52]. Systemic features, such as cachexia, diabetes, or jaundice, can further modulate pain perception, but are not assessed by existing instruments [46].

The COPRAC Delphi study defines abdominal complaints as one of 8 core outcomes to include in the assessment of PDAC patients, prioritized by clinicians and patients across six centers distributed globally [53]. However, even this consensus core outcome set lacks specificity regarding pain type, location, and temporal variation. The absence of disease-specific instruments for PDAC pain assessment limits targeted treatment. Visceral and neuropathic nociception often require different treatment modalities, and comprehensive assessment might therefore enable the clinician to move away from empirical therapy and toward an individualized, mechanism-oriented treatment.

### Quantitative sensory testing and mechanistic profiling

Quantitative sensory testing (QST) has recently been applied to PDAC to evaluate somatosensory function and serve as a proxy for central pain processing. Using standardized stimuli, including pressure, pinprick, and conditioned pain modulation, QST detects sensory gain or loss patterns that reflect peripheral and central sensitization [14, 54]. Studies using the pancreatic-QST protocol [55] have demonstrated significant abnormalities in patients with PDAC compared with healthy controls, including lower pressure-pain thresholds at the pancreatic dermatomes, enhanced temporal summation, and reduced conditioned pain modulation [14]. These findings suggest that many patients develop segmental and widespread hyperalgesia and are consistent with results from studies in chronic pancreatitis, where pain has many comparable characteristics [55]. Such alterations are not evident when pain is assessed on conventional intensity scales or even multidimensional scales and may therefore serve as a supplement to these instruments. However, at present, QST remains primarily a research tool and is not part of routine clinical pain assessment in most centers. Although standardized protocols allow testing to be performed reproducibly by trained personnel, results can vary between sites, and local reference values are needed. Broader clinical implementation is further limited by the need for specialized equipment, staff training, dedicated testing time, and the lack of reimbursement in routine clinical practice. In PDAC, findings may also vary with tumor stage, cachexia, opioid exposure, or systemic inflammation.

A combined subjective–objective approach could enable subclassification of PDAC pain into phenotypes based on pain type and characteristics, with implications for individualized therapy. Incorporating QST and multidimensional outcome measures into clinical trials and a palliative framework would represent a significant step toward mechanism-based pain management in PDAC.

## Pharmacological therapy

### Principles and goals, opioids, and special considerations

There are very few studies specifically addressing pharmacological pain management in PDAC; therefore, current recommendations are largely extrapolated from the treatment of cancer-related pain in general. The primary goal is optimal pain control with minimal adverse effects while preserving quality of life [56].

**Fig. 2:**
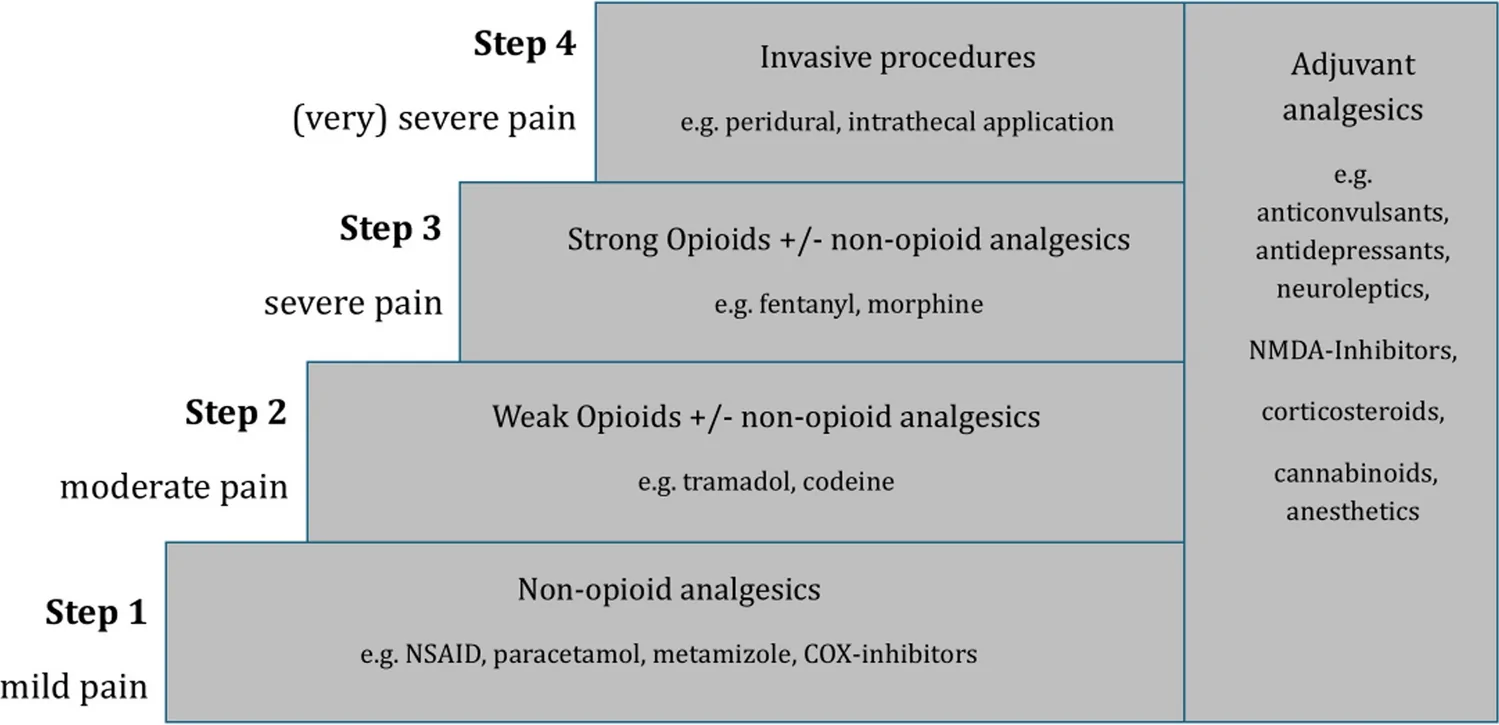
Stepwise approach to pharmacological pain therapy in PDAC, analogous to the WHO analgesic ladder with extension. *NSAID* non-steroidal anti-inflammatory drugs, *COX* cyclooxygenase, *NMDA* N-methyl-D-aspartate

The World Health Organization (WHO) originally proposed a three-step analgesic ladder, recommending non-opioids for mild pain, weak opioids for moderate pain, and strong opioids for severe pain (Fig. 2). To reduce opioid doses, they should be combined with non-opioid analgesics whenever possible [52, 57]. Acetaminophen is preferred—where available, metamizole represents an alternative [9]. Non-steroidal anti-inflammatory drugs (NSAIDs) may also be used, but not first-line, due to their adverse effect profile. In opioid-naïve patients, treatment should be initiated with a weak opioid (e.g., tramadol) or a low-dose potent opioid (e.g., morphine or oxycodone) with a rapid onset of action [56]. Increasing evidence suggests that low-dose strong opioids, such as morphine, may be a valid alternative to step 2 therapy in the ladder. Consequently, recent WHO recommendations have moved away from a rigid three-step model toward a more individualized treatment approach [57, 58]. Advantages of strong opioids include faster and more comprehensive pain control and, in some cases, lower costs, while higher rates of adverse effects are discussed as potential disadvantages [58].

In clinical practice, weak opioids are used relatively infrequently in PDAC. A multicenter cross-sectional study showed that only 9% of patients with painful disease received weak opioids, compared with 24% treated with strong opioids [5]. The overall low opioid prescription rates indicate that more than half of patients were undertreated. Pain undertreatment in PDAC is likely multifactorial. In principle, barriers, including patient-related factors, should be addressed through regular standardized pain assessments and comprehensive patient education. This approach aims to overcome prejudices against opioid therapy and to emphasize the patient’s active role in pain management [5, 59]. There is no evidence for clear superiority of any specific opioid [56, 60]. The selection depends on patient characteristics, pain features, comorbidities, and physician experience, and guidelines for opioid use in general can be followed [61].

Analgesics should preferably be administered orally at fixed time intervals according to a predefined schedule, ensuring that the next dose is given before the effect of the previous dose diminishes [57, 59]. If oral administration is not feasible, parenteral routes, such as transdermal, subcutaneous, or intravenous administration, are available. Intravenous administration is particularly suitable for rapid pain control [59]. When switching from oral to intravenous or subcutaneous routes, the higher relative analgesic potency of parenteral administration (approximately 1:2 or 1:3) must be considered [52]. If fast-acting opioids are required several times daily, conversion to baseline therapy with a sustained-release formulation is recommended [56]. Breakthrough pain can be treated with additional fast-acting opioids every 3–4 h, with the rescue dose corresponding to approximately 10–20% of the total daily dose. Frequent need for rescue medication should prompt individual upward titration of baseline therapy.

For unpredictable, severe breakthrough pain and in patients having problems with swallowing or malabsorption, transmucosal fentanyl formulations (buccal, sublingual, or intranasal) are particularly effective [9, 59]. If adequate pain control is not achieved or adverse effects are intolerable, opioid rotation should be considered [52]. In patients with hepatic dysfunction, opioids should be administered at lower doses or with prolonged dosing intervals [56]. Morphine and hydromorphone are metabolized independently of cytochrome P450 enzymes and, therefore, have a lower risk of CYP-mediated drug interactions [56]. In patients with impaired renal function (glomerular filtration rate < 30 ml/min), opioid accumulation may lead to opioid-induced neurotoxicity. In such cases, switching to transdermal fentanyl or buprenorphine, or to methadone in experienced hands, is recommended [56, 59]. In paradoxical worsening of pain under opioid therapy (opioid-induced hyperalgesia or narcotic bowel), dose reduction or opioid rotation may be beneficial [62, 63].

### Adjuvant analgesics, and supportive and combination strategies

Adjuvant analgesics are used to enhance the effects of opioids, especially when there is an insufficient response to opioids. Clinically, they can be grouped into anticonvulsants like gabapentinoids, antidepressants like selective serotonin reuptake inhibitors (SSRIs), serotonin/norepinephrine reuptake inhibitors (SNRIs) and tricyclic antidepressants (TCAs), as well as neuroleptics, corticosteroids, and local anesthetics/topical agents [64]. They are primarily used to reduce visceral and neuropathic pain. Although most of these agents have not been systematically evaluated for pain associated with PDAC, treatment selection is often guided more by the individual pain phenotype than by the disease label itself, as phenotypic variability may be greater between patients than between pain syndromes [65, 66]. Accordingly, the use of gabapentinoids and antidepressants in PDAC is largely extrapolated from the broader cancer pain and neuropathic pain literature rather than from PDAC-specific trials [67].

In broader cancer-related neuropathic pain settings, the most commonly used anticonvulsants, gabapentin and pregabalin, have been associated with improved pain control [68] and quality of life when combined with opioids. Furthermore, they reduce the incidence of nausea and vomiting; however, data specifically in PDAC remain sparse [69, 70]. While gabapentin is effective against neuropathic pain in chemotherapy-associated mucositis, pregabalin has been shown to be superior to transdermal fentanyl in cancer-related neuropathic pain. In a randomized prospective study by Raptis et al., patients treated with pregabalin reported better pain response, higher treatment satisfaction, and fewer side effects and treatment discontinuations [71].

When prescribing antidepressants, drug interactions must be checked, as the drugs often inhibit hepatic drug metabolism by inhibition of cytochrome P450 enzymes, especially CYP2D6. Additionally, steroids can contribute to pain reduction in neuropathic and bone pain. By inhibiting prostaglandin synthesis, they reduce vascular permeability, leading to lower pain intensity through their anti-inflammatory effects. This has resulted in an improvement in sleep quality and daily activity [72]. For severe neuropathic pain, a combination of opioids and the N-methyl-D-aspartate-receptor antagonist ketamine can be used, although the evidence is scarce. The best efficacy is observed with patient-controlled epidural administration, but the mode of administration is still not standardized. Maximum response was achieved between 2 days and 2 weeks after administration. This resulted in lower morphine consumption without evidence of sedative side effects [73]. Depressive symptoms were also less frequent. This approach is primarily used in stage IV PDAC. Ketamine can also be effective in the treatment of opioid-induced hyperalgesia, which can especially occur with high opioid doses and fast-acting medications [74].

In addition to visceral and neuropathic pain, PDAC-associated pain can also arise from exocrine pancreatic insufficiency. This may lead to postprandial pain and bloating. Early enzyme replacement therapy can normalize digestion. However, its effect on pain in PDAC is not proven [56].

In correlation to increasing legalization of cannabinoids, more patients with cancer are using cannabinoids and corresponding derivatives to treat cancer- and therapy-associated symptoms like nausea, low appetite, and poor sleep. Regarding the significance of cannabinoids in pain therapy, the literature presents conflicting information. Two randomized-controlled trials in patients with cancer-associated pain demonstrated the efficacy of low and medium doses of nabiximols [75] or tetrahydrocannabinol:cannabidiol extract, whereas another study failed to detect a significant reduction in neuropathic pain with nabiximols [76, 77]. In conclusion, there is currently no strong evidence for the use of cannabinoids in pain therapy in PDAC [78].

In addition to the combination of non-opioid analgesics with low- or high-potency opioids, as established within the WHO analgesic ladder, combination therapy with co-analgesics might be implemented early in the course of the disease. This may allow lower opioid doses to be sufficient for symptom control and reduce opioid-related side effects. The choice of co-analgesics depends on the patient’s comorbidities, adverse effects, and desired side effects like sleep and anxiolytic action. Patients with anxiety and depression may benefit from the additional antidepressant effects of SNRIs or TCAs.

## Neurolysis and irradiation therapy

Neurolysis is an established interventional strategy for pain management. In patients with PDAC, pain that is frequently refractory to standard analgesics may occur due to tumor invasion of the celiac plexus. As early as 1993, Lillemoe et al. described significantly reduced pain scores after performing chemical splanchnicectomy intraoperatively in PDAC patients using 50% alcohol injected into the celiac plexus. Pain assessments were performed every two months, and patients who received chemical splanchnicectomy reported greater pain relief than those managed with standard analgesics alone. However, the effects of alcohol splanchnicectomy were not permanent. More than 60% of patients with significant preoperative pain, relieved by an alcohol block, had moderate-to-severe pain recurring before death [79].

Nowadays, CPN is commonly performed as a minimally invasive procedure for pain management in PDAC patients, particularly those with unresectable disease and refractory abdominal pain. The technique involves percutaneous computed tomography (CT)- or endoscopic ultrasound (EUS)-guided injection of a neurolytic agent, most commonly absolute ethanol, into the celiac plexus to disrupt visceral afferent pain transmission from the pancreas. A recent meta-analysis by Koulouris et al. [48] showed that EUS-CPN is effective in providing pain relief, with response rates of approximately 68% at 2 weeks and 53% at 4 weeks post-intervention, and pain relief typically lasts for a median of 2–3 months [80]. In contrast, a randomized study by Kanno et al. showed no differences in pain or quality of life between EUS-CPN and conventional treatment with strong opioids [81]. Repeat procedures may be necessary, but usually do not have the same pain relief. Pain reduction is mostly partial, with many patients continuing to require opioids, albeit typically at stable or reduced doses. However, improvement in quality of life has not been seen [20]. Consistent with these findings, a recent systematic review and meta-analysis reported no significant difference in pain reduction between celiac plexus neurolysis and standard pharmacological therapy up to 12 weeks, indicating comparable analgesic efficacy, albeit with possible complementary effects [82]. Accordingly, celiac plexus neurolysis and related neurolytic interventions are best considered components of multimodal pain management and are generally used in combination with ongoing pharmacological therapy rather than as stand-alone treatment [50, 83]. Significant predictors of poor response to EUS-CPN for pancreatic cancer pain are invisible celiac ganglia, distant metastasis, and direct invasion of the celiac plexus, although tumors located in the body/tail of the pancreas and patients receiving bilateral treatment tend to be associated with a good response. Additionally, there was no significant difference in analgesic efficacy observed among the three techniques studied (central injection, bilateral injection, and celiac ganglia neurolysis), though central injections seem to have less severe complications [48].

Regarding CT-guided CPN, Dong et al. found that neurolytic splanchnic nerve block with CT-guided alcohol injection via transdiscal or paraspinal routes provides significant short-term pain relief in patients with reductions in pain scores sustained for up to 3 months post-intervention. Both transdiscal and paraspinal approaches were equally effective for pain control, and both led to decreased opioid consumption [20]. Yoon et al. compared EUS-CPN and percutaneous CT-guided celiac neurolysis (PCN), finding no statistically significant differences in pain reduction, opioid use, quality of life, patient satisfaction, or adverse event rates between EUS-CPN and PCN [83]. Both methods were found to be similarly effective and safe [83].

Most interestingly, several studies examined the potential impacts of celiac neurolysis on the survival of PDAC patients. In this context, the early study by Lillemoe et al. described a significant improvement in survival after open chemical splanchnicectomy in patients with preoperative pain, hypothesizing that patients with chronic pain may be more likely to experience malnutrition and immobility with life-threatening complications, such as pneumonia and worsening survival [79]. This was supported by a double-blinded, randomized, placebo-controlled study by Staats et al., concluding that neurolysis could prolong life even in patients with unresectable cancer [84]. In contrast, a retrospective study by Oh et al. showed no significant effect of CPN on survival in patients with unresectable pancreatic cancer, though the timing of CPN after diagnosis was inconsistent [85]. In addition to that, a meta-analysis of Ye et al. concluded that celiac neurolysis and splanchnicectomy do not improve overall survival in patients with unresectable pancreatic cancer. Subgroup analysis revealed that celiac neurolysis or splanchnicectomy may even be associated with worse survival in patients with stage III pancreatic cancer, but not in those with stage IV disease. Dong et al. also found that neurolytic splanchnic nerve block could shorten survival in general, and subanalysis showed that the effect was mainly seen in stage IV disease [20, 86]. These data suggest that while CPN procedures are effective for pain control, the impact on survival remains controversial.

Likewise, the timing of neurolysis remains controversial as well. Currently, neurolysis is often used as a form of rescue therapy when systemic analgesic therapy is inadequate. Oliveira et al. suggested that neurolysis should be considered earlier for the management of cancer pain [87]. Additionally, Wyse et al. stated that CPN should be considered at the time of diagnosis and staging, because the earlier the neurolysis is performed, the more the patient can benefit [88]. On the other hand, a randomized study showed that controlling pain with medication and performing the celiac block later seems to be the most effective [89]. The probability of patients remaining completely pain-free after CPN diminishes with increasing survival time, where the tumor and metastasis involve areas of somatic innervation such as the peritoneum or diaphragm. The optimal therapeutic window may therefore be when analgesic treatment becomes insufficient, but before the very late stages of the disease, so the effect is maximal when pain intensity is highest and of pancreatic origin only [9]. Life expectancy, and the extent and rate of disease progression should also be considered in individual patients.

Besides direct injections of neurolytic or neurotoxic agents, irradiation therapy approaches for pain management have been proposed, including conventional short-course palliative radiotherapy, stereotactic body radiotherapy, and EUS-guided irradiation with injection of iodine-125 seeds. In a retrospective study using the ladder, Tung et al. showed that radiation therapy for pain management—although nowadays rarely used—was associated with meaningful pain reduction [90]. In line with that, Lawrence et al. evaluated celiac plexus radiosurgery for PDAC patients experiencing refractory retroperitoneal pain syndrome. Eligible patients had moderate-to-severe pain. The intervention was a single fraction of 25 Gy external-beam photon radiotherapy targeted at the celiac plexus. At 3 weeks post-treatment, 53% of evaluable patients achieved at least partial pain response and improvement in quality of life [91]. The maximal effect occurred at approximately 1 month post-treatment. However, pain levels tend to increase again after this period, indicating that the analgesic benefit of SBRT is not sustained [92].

Furthermore, the PAINPANC trial evaluated short-course palliative radiation therapy (24 Gy in 3 weekly fractions) in patients with moderate-to-severe, medication-refractory pain. This study demonstrated a rapid and clinically relevant reduction in pain severity, a significant reduction in opioid requirements, and an improvement in global quality of life, with mostly mild toxicity [92].

Wang et al. showed that EUS-guided implantation of 2–6 iodine-125 seeds directly into the celiac ganglion led to significant pain relief and reduction in opioid consumption beginning two weeks after the procedure [93]. Zhang et al. found that EUS-CPN provides more rapid pain relief, while EUS-125I provides more durable and extensive pain control. The study also noted a difference in median survival time between the groups, suggesting a potential survival benefit with EUS-125I, though the mechanism and clinical significance require further investigations [94].

In a very recent preclinical model, Thiel et al. found that PDAC cells are able to reprogram single neurons to support tumor progression and immune evasion. Cancer-driven neuronal reprogramming contributes to further reshaping of the tumor microenvironment. In a mouse model, pharmacological denervation of PDAC-innervating neurons led to a pro-inflammatory microenvironment and enhanced the efficacy of immune checkpoint inhibitors. Furthermore, Thiel et al. observed that chemical denervation, synergized with chemotherapy, led to a reduction in tumor size compared with animals that received chemotherapy only [95]. It remains to be shown whether these intriguing preclinical findings can be translated into a therapeutic benefit of multimodal concepts, including neurolysis, in PDAC patients.

In conclusion, neurolysis performed by CT- or EUS-guided injections or by irradiation represents an effective tool for pain management in PDAC, but available studies show considerable variations in onset, extent, and durability of its analgesic efficacy. Irradiation is less invasive and avoids the procedural risks of direct injections. The impact of neurolysis on survival remains controversial and warrants further investigation. In patients requiring high-dose opioids, the selection of different modalities for neurolysis should be individualized based on patient anatomy, disease stage, and institutional expertise.

## Conclusion

The pathophysiology of pain in PDAC is complex, multifactorial, and poorly understood. The combined effects of tumor growth and spread, perineural invasion, neuroimmune interactions, peripheral nerve remodeling, and central nervous system sensitization, along with additional modulation by comorbid conditions (such as anxiety or depression) and treatment-related toxicity, drive PDAC pain. Pain in PDAC is most commonly assessed using unidimensional intensity scales, such as numeric or visual analog scales, which measure severity but fail to capture disease-specific features, temporal variation, or underlying pain mechanisms. Multidimensional tools like the Brief Pain Inventory assess functional interference, but do not address key PDAC-specific characteristics such as postprandial worsening, neuropathic descriptors, or mixed visceral-neuropathic pain patterns. As a result, pain in PDAC is poorly characterized and frequently undertreated, particularly in older and newly diagnosed patients, and is closely linked with broader functional and psychological distress. Pancreatic QST has been utilized to phenotype pain in PDAC patients in research settings and shows promise for the future. While the use of the WHO analgesic three-step ladder is advocated for the pharmacologic management of painful PDAC, the majority of patients are treated with strong opioids. The efficacy of neuromodulators has not been established for this indication. Endoscopic and percutaneous celiac plexus neurolysis are frequently utilized to treat PDAC pain, but the unpredictability and durability of response, as well as possible reduced survival associated with celiac ganglia injection, especially in patients with advanced disease, have tempered enthusiasm. Novel interventional approaches continue to be evaluated for the treatment of pain in PDAC but are not ready for routine clinical use.
